# Epidemiology of recurrent seizure disorders and epilepsy in cats under primary veterinary care in the United Kingdom

**DOI:** 10.1111/jvim.15881

**Published:** 2020-09-25

**Authors:** Dan Gerard O'Neill, Stephanie A. Phillipps, Jordon R. Egan, David Brodbelt, David B. Church, Holger A. Volk

**Affiliations:** ^1^ Royal Veterinary College—Veterinary Epidemiology and Public Health Hertfordshire United Kingdom; ^2^ University of Liverpool Institute of Veterinary Science Ringgold Standard Institution—Department of Small Animal Clinical Science Merseyside United Kingdom; ^3^ Royal Veterinary College—Clinical Sciences and Services Hertfordshire United Kingdom; ^4^ University of Veterinary Medicine Hannover, Department of Small Animal Medicine and Surgery Hannover Germany

**Keywords:** feline, first opinion, general practice, pedigree, purebred, VetCompass

## Abstract

**Background:**

Little epidemiological evaluation of recurrent seizure disorders in cats currently exists in veterinary literature.

**Objectives:**

To report the prevalence and risk factors for recurrent seizure disorders (RSD) and epilepsy in cats presented to primary care veterinary practices in the United Kingdom (UK).

**Animals:**

A total of 285 547 cats under veterinary care during 2013 presenting to 282 primary care clinics in the UK.

**Methods:**

Cohort study using multivariable logistic regression modeling for risk factor analysis.

**Results:**

There were 458 confirmed RSD cases, giving a 1‐year period prevalence of 0.16% (95% confidence interval [CI], 0.15‐0.18). A subset of 114 (24.89%) cases was recorded as having epilepsy, giving a 1‐year period prevalence of 0.04% (95% CI, 0.03‐0.5). Increasing age was significantly associated with increasing odds of RSD. Breed, sex, neuter status, and body weight were not associated with RSD. Epilepsy was most frequently diagnosed in adult to middle‐aged cats. Cats aged 3.0 to <6.0 years had 3.32 times higher odds of epilepsy diagnosis compared to cats <3.0 years of age. Insured cats were more likely to be diagnosed with epilepsy compared to noninsured cats.

**Conclusions and Clinical Importance:**

Although less common than in dogs, RSD and epilepsy still comprise an important disorder group in the UK cat population. Aging is a significant risk factor for these disorders in cats.

**Abbreviations**
ASD: antiseizure drug
CI: confidence intervals
CSF: cerebrospinal fluid
EPR: electronic patient record
IQR: interquartile range
MRI: magnetic resonance imaging
OR: odds ratio
ROC: receiver operating characteristic
RSD: recurrent seizure disorders; UK, United Kingdom

## 1 INTRODUCTION

Neurological disorders are common in cats and are among the leading causes of mortality in the pet feline population.^1^, ^2^ Of these neurological disorders, seizures are a common reason for cats to be presented to a veterinary neurologist, with the prevalence of seizures in referral populations of cats reported as 2.1% in Germany^3^ and 3.5% in Austria.^4^ Cats with seizures represent an important and challenging presentation for the general veterinarian, and these conditions have the potential to cause great concern and emotional distress for owners.^5^ Recurrent seizure disorders (RSD) including epilepsy have been the topic of extensive veterinary research in companion animal species over the last 75 years, but almost exclusively based on caseloads from referral populations and teaching hospitals. However, the reliability of generalization from these secondary and tertiary care subsets to the wider cat population has been questioned.^6^ Research on RSD in dogs utilizing datasets from primary practice electronic patient records (EPR) have reported a 1‐year period prevalence for seizure occurrence in dogs of 0.82% and for suspected idiopathic epilepsy of 0.62% to 0.75%.^7^, ^8^ However, to date, no similar studies have described RSD in cats.

Recurrent seizure disorders in cats represent a diagnostic challenge for several reasons. Seizure type in cats is often atypical.^9^, ^10^, ^11^ Cats frequently present with focal epileptic seizures,^12^, ^13^ which can be misleading for both owners and practitioners and may be mistaken for other paroxysmal events.^14^, ^15^ Second, debate about the true prevalence and clinical relevance of spontaneous genetic epilepsy in cats is ongoing. Traditionally, genetic epilepsy was believed to be rare in cats,^12^, ^16^ but a genetic basis since has been identified in a laboratory cat colony and now is considered an important differential diagnosis in cats.^11^, ^17^, ^18^

Confusion about diagnosis of epilepsy in both cats and dogs is exacerbated by inconsistent use of the diagnostic terms “epilepsy” and “idiopathic epilepsy” in the veterinary literature.^16^, ^19^ The International Veterinary Epilepsy Task Force (IVETF) addressed these issues for dogs, but not for cats.^5^ Fluidity of these definitions has led some authors to adopt the term “epilepsy of unknown cause” to describe RSD with a clinically unremarkable diagnostic evaluation, with the aim of maintaining a neutral stance on whether these disorders are truly “idiopathic” or if the diagnostic techniques available in veterinary medicine are currently unable to elucidate the etiology.^20^ Although concise definitions that can be universally agreed on undoubtedly will aid future research efforts by use of precise terms to differentiate specific etiologies, overzealous definitions should not detract from insight that is currently available through more generalized use.^5^ Defining disorders based on their phenotypic signature (eg, RSD) rather than reliance on uncertain biomedical terms (eg, epilepsy) applied by clinicians may offer increased reliability for research into disorder frequency.^21^, ^22^

Evidence relevant to the general population of animals under primary veterinary care should be derived from the general population of animals under primary veterinary care.^23^ Consequently, several large projects are now underway that aim to merge anonymized clinical data from primary care veterinary clinics into single databases for research.^24^, ^25^, ^26^, ^27^ Research using primary care veterinary clinical records benefits from contemporaneous recording at the time of the clinical events by veterinary professionals across the spectrum of species and disorders recorded during their care.^28^ We aimed to estimate the prevalence of RSD and epilepsy in the wider cat population under primary veterinary care in the United Kingdom (UK) and to evaluate demographic risk factors for their occurrence. A secondary aim was to explore risk factors associated with diagnosis of epilepsy among the subset of cats with RSD. This information could promote understanding of the clinical rationale applied by clinicians when assigning biomedical diagnostic terms to neurological clinical cases in cats.

## 2 METHODS

The VetCompass Programme collates deidentified EPR data from primary care veterinary practices in the UK for epidemiological research.^26^ VetCompass collects information fields that include clinic attended, species, breed, date of birth, sex, neuter status, insurance status, body weight, and clinical information from free‐form text clinical notes and summary diagnosis terms (VeNom codes^29^), as well as treatment and deceased status with relevant dates.

A cohort study of cats attending VetCompass practices was used to estimate the prevalence and risk factors for RSD and epilepsy. The study population included all cats under veterinary care within the VetCompass database from January 1, 2013 to December 31, 2013. Recurrent seizure disorder cases required evidence that they met the case definition during 2013. “Under veterinary care” was defined as having at least 1 EPR recorded from January 1st to December 31st 2013 or at least one EPR both before and after 2013. With no prior reported prevalence values for RSD in cats available, sample size estimation was explored using both 0.25% and 0.10% expected prevalence as conservative estimates, substantially lower than the 0.82% reported in dogs in the UK.^8^ Sample size calculations estimated that 38 176 cats were required to estimate the prevalence of a disorder with a 0.25% expected prevalence and 15 321 cats were required to estimate the prevalence of a disorder with a 0.10% to a precision of 0.05% at a 95% confidence level from a UK cat population of 8 million cats.^30^, ^31^ Ethical approval was granted by the Royal Veterinary College Ethics and Welfare Committee (2016/BSc 20172).

Given an a priori awareness of the likely inconsistency of clinical diagnoses applied to seizure disorders in cats,^16^, ^19^ we applied a phenotypic signature approach to searching and assigning the RSD cases that were included in the analysis.^22^ Case inclusion criteria for RSD required that at least 1 of the following criteria applied during 2013: at least 2 episodes of seizure events with a minimum of 24 hours between the first and final events, a final diagnosis of epilepsy or synonym (eg, epileptic) was recorded in the EPR, or was prescribed an antiseizure drug (ASD) to manage a seizure‐related disorder (based on an assumption that ASD treatment was unlikely to be prescribed for a solitary seizure event). Cats with only extracranial reactive seizures (ie, seizures secondary to a primary condition external to the brain, such as hepatic encephalopathy, electrolyte imbalances, toxicity, or agonal seizuring) were excluded. The case definition for epilepsy required that a final diagnosis of epilepsy or synonym (eg, epileptic) was recorded in the EPR by the primary care veterinary team. Case finding involved initial screening of all EPR for candidate RSD cases using a bank of search terms including epil*, seiz*, seizure~2, had 1 fit, had 2 fits, phenob*, epiphen~2, anti‐epil*, anti‐convuls*, potassium bromide, KBr, levetira*, keppra, zonisam*, 2 fits, short fit, lyrica, pregabalin, and gabapentin. Candidate cases were randomized and the clinical notes of all candidates were reviewed to evaluate for case inclusion. Additional data were extracted on confirmed cases to describe the date of the first recorded seizure for the RSD overall, and whether the case was diagnosed with epilepsy by the first opinion practitioner. Confirmed RSD cases were grouped as “RSD cases” and all remaining study cats were grouped as “noncases.”

A “purebred” variable categorized all cats of recognizable breeds as “purebred” and the remaining cats as “crossbred.” A “breed” variable included individual breeds that had at least 1 RSD case or that were represented by at least 1000 study animals, a grouped category of all remaining purebreds, and a general grouping of crossbred cats. This approach was taken to allow focus on commonly affected breeds and on common breeds, and to facilitate statistical power for the individual breed analyses.^32^ *A* “neuter” variable described the status of the cat (neutered or intact) recorded at the final EPR. An “insurance” variable described whether a cat was insured at any point during the study period. An “age” variable categorized age (years): <3.0, 3.0‐<6.0, 6.0‐<9.0, 9.0‐<12.0, 12.0‐<15.0, ≥15.0, not recorded. Age (years) was calculated for RSD cases at the first recorded seizure event and for noncase cats at December 31st, 2013 (the latest date the cat was known to be seizure‐free). This approach was taken so that the age results would reflect the odds of “becoming” a case rather than the odds of “being” a case. An “adult body weight” variable categorized adult body weight: <3.0 kg, 3.0‐<4.0 kg, 4.0‐<5.0 kg, 5.0‐<6.0 kg, ≥6.0 kg, not available. Adult body weight described the maximum body weight recorded during the study period for cats >6 months of age. “Dominant color” defined a categorical variable that included all colors that were recorded as comprising some or all of the coloration for at least 5000 study animals along with a grouped category of all remaining less‐common colors. The dominant color ascribed for individual cats was determined by the first color term used to describe the cat. “Self‐color” defined a binary variable describing whether the cat was recorded as self‐colored (ie, with only a single solid color) or not.^33^

After data checking and cleaning in Excel (Microsoft Office Excel 2013, Microsoft Corp.), analyses were conducted using Stata Version 13 (Stata Corporation). The 1‐year period prevalence describes the proportion of all study animals recorded with the disorder during a specified 12‐month period, which was 2013 in the current study.^34^ The 95% confidence interval (CI) estimates were derived from standard errors, based on approximation to the binomial distribution.^35^ Descriptive statistics characterized the purebred status, breed, sex, color, neuter status, insurance, age and adult body weight for the RSD cases and noncases. The chi squared test was used for statistical comparison between categorical variables.^35^

### 2.1 Risk factors for RSD in the overall population of cats

Binary logistic regression modeling was used to evaluate univariable associations between risk factors (purebred, breed, adult body weight, age, sex, neuter, insurance, dominant color, self‐color), and RSD. Risk factors with liberal associations in univariable modeling (*P* < .2) were taken forward for multivariable evaluation. Model development used manual backwards stepwise elimination. Pair‐wise interaction effects were evaluated for the final model variables. Confounding effects from dropped variables were assessed by individual reintroduction to the final model. The likelihood ratio test was used to compare a random effects model with clinic entered as a random effect against the nonrandom effects model with *P* < .05 cut‐off used for selection of the random effects model. The Hosmer‐Lemeshow test statistic^36^ and the area under the receiver operating characteristic (ROC) curve were used to evaluate model fit.^34^ Statistical significance was set at *P* < .05.

### 2.2 Risk factors for epilepsy in the overall population of cats

These methods were repeated to evaluate risk factors associated with epilepsy in the overall population after dropping RSD cases that were not classified as epilepsy.

### 2.3 Risk factors for epilepsy among cats with RSD

These methods also were applied to explore risk factors associated with diagnosis of epilepsy among the subset of cats that were recorded with RSD.

## 3 RESULTS

The study population consisted of 285 547 cats under veterinary care during 2013 attending 282 primary care clinics in the UK. There were 1497 cats identified as candidate RSD cases. All candidate cases were checked to confirm 458 (30.59% of the candidates) as RSD cases, giving an overall 1‐year period prevalence for RSD in cats in the UK of 0.16% (95% CI, 0.15‐0.18). Breeds with the highest RSD prevalence were Foreign (1.89% of the breed affected; 95% CI, 0.05‐10.07) and Burmilla (0.84%; 95% CI, 0.02‐4.59). The prevalence of RSD did not differ between crossbred cats (0.17%; 95% CI, 0.15‐0.18) and purebred cats (0.13%; 95% CI, 0.09‐0.18, *P* = .165; Table 1).

**TABLE 1:** Prevalence of recurrent seizure disorders (RSDs) in common cat breeds under primary veterinary care in the UK

Breed	No. cats in study	No. cases	Prevalence %	95% CI
Foreign	53	1	1.89	0.05‐10.07
Burmilla	119	1	0.84	0.02‐4.59
Exotic	450	2	0.44	0.05‐1.60
Birman	1150	5	0.43	0.14‐1.01
Siberian	236	1	0.42	0.01‐2.34
Burmese	1500	4	0.27	0.07‐0.68
Russian	507	1	0.20	0.01‐1.09
Norwegian Forest	568	1	0.18	0.00‐0.98
Crossbred	252 349	417	0.17	0.15‐0.18
British Short Hair	5283	8	0.15	0.07‐0.30
Persian	3314	5	0.15	0.05‐0.35
Bengal	3344	4	0.12	0.03‐0.31
Maine Coon	1969	2	0.10	0.01‐0.37
Ragdoll	2905	2	0.07	0.01‐0.25
Siamese	2503	1	0.04	0.00‐0.22
British Blue	1507	0	0.00	0.00‐0.24
Other purebreds	3664	0	0.00	0.00‐0.10
Breed not recorded	4123	3	0.07	0.02‐0.21
Overall	285 547	458	0.16	0.15‐0.18

Abbreviation: CI, confidence interval.

Of the 458 RSD cases, 114 (24.89%) were recorded with epilepsy by the first opinion veterinary practitioners, giving an overall 1‐year period prevalence for first opinion‐classified epilepsy in cats in the UK of 0.04% (95% CI, 0.03‐0.05). In addition, the possibility of epilepsy was discussed in the clinical records of an additional 69 RSD cases without confirmation recorded in the notes.

**FIGURE 1:**
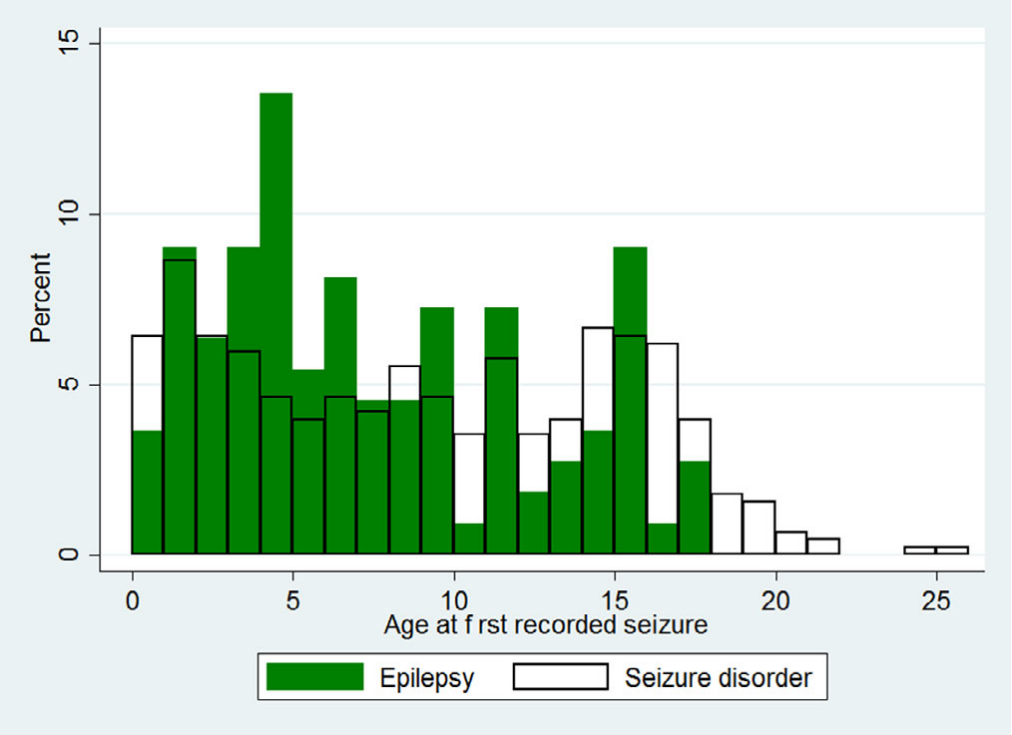
Age (years) at the first recorded seizure event for cats under primary veterinary care in the UK diagnosed with recurrent seizure disorders (RSD) (N = 458) and epilepsy (N = 114)

Of the RSD cases with complete data available for that variable, 38/455 (8.4%) were purebred, 240/456 (52.6%) were female, 285/324 (88.0%) were neutered, 88/161 (54.7%) were insured, and 235/448 (52.5%) were self‐colored. The most common colors of RSD cases (color information available on 448 cats) were black (n = 182, 40.6%) and tabby (n = 94, 21.0%; Table 2). The median adult body weight for RSD cats was 4.4 kg (interquartile range [IQR], 3.6‐5.5; range, 2.2‐9.6). The median age at first recorded seizure event for RSD cases overall was 8.9 years (IQR, 3.5‐14.2; range, 0.2‐25.1). The median age at the first recorded seizure event for the subset of RSD cases recorded with epilepsy was 6.4 years (IQR, 3.5‐11.3; range, 0.2‐18.0) (Figure 1).

**TABLE 2:** Descriptive and univariable logistic regression results for risk factors associated with diagnosis of recurrent seizure disorders (RSD) in cats under primary veterinary care in the UK

Variable	Category	Case no. (%)	Noncase no. (%)	Odds ratio	95% CI	Category *P*‐value	Variable *P*‐value
Purebred status	Crossbred	417 (91.1)	251 932 (88.4)	Base			.1
	Purebred	38 (8.3)	29 034 (10.2)	0.79	0.57‐1.10	.2	
	Unrecorded	3 (0.7)	4123 (1.5)	0.44	0.14‐1.37	.2	
Common breeds	Crossbred	417 (91.1)	251 932 (88.4)	Base			.2
	Bengal	4 (0.9)	3340 (1.2)	0.72	0.27‐1.94	.5	
	Birman	5 (1.1)	1145 (0.4)	2.64	1.09‐6.38	.03	
	British Blue	0 (0.0)	1507 (0.5)	…			
	British Short Hair	8 (1.8)	5275 (1.9)	0.92	0.45‐1.85	.8	
	Burmese	4 (0.9)	1496 (0.5)	1.62	0.60‐4.33	.3	
	Burmilla	1 (0.2)	118 (0.0)	5.12	0.71‐36.74	.1	
	Exotic	2 (0.4)	448 (0.2)	2.70	0.67‐10.85	.2	
	Foreign	1 (0.2)	52 (0.0)	11.62	1.60‐84.24	.02	
	Maine Coon	2 (0.4)	1967 (0.7)	0.61	0.15‐2.47	.5	
	Norwegian Forest	1 (0.2)	567 (0.2)	1.07	0.15‐7.60	1	
	Other purebreds	0 (0.00)	3664 (1.3)	…			
	Persian	5 (1.1)	3309 (1.2)	0.91	0.38‐2.21	.8	
	Ragdoll	2 (0.4)	2903 (1.0)	0.42	0.10‐1.67	.2	
	Russian	1 (0.2)	506 (0.2)	1.19	0.17‐8.51	.9	
	Siamese	1 (0.2)	2502 (0.9)	0.24	0.03‐1.72	.2	
	Siberian	1 (0.2)	235 (0.1)	2.57	0.36‐18.37	.4	
	Unrecorded	3 (0.7)	4123 (1.5)	0.44	0.14‐1.37	.2	
Self‐color	Single	235 (51.3)	137 752 (48.3)	Base			.08
	Multiple	213 (46.5)	136 251 (47.8)	0.92	0.76‐1.10	.4	
	Unrecorded	10 (2.2)	11 082 (3.9)	0.53	0.28‐1.00	.05	
Dominant color	Black	182 (39.7)	108 551 (38.1)	Base			.43
	Tabby	94 (20.5)	52 152 (18.3)	1.08	0.84‐1.38	.6	
	Ginger	35 (7.6)	25 902 (9.1)	0.81	0.56‐1.16	.24	
	Tortoiseshell	40 (8.7)	24 508 (8.6)	0.97	0.69‐1.37	.9	
	Gray	24 (5.2)	16 182 (5.7)	0.88	0.58‐1.35	.6	
	White	28 (6.1)	15 357 (5.4)	1.09	0.73‐1.62	.7	
	Blue	10 (2.2)	6219 (2.2)	0.96	0.51‐1.82	.9	
	Other color	35 (7.6)	25 132 (8.8)	0.83	0.58‐1.19	.32	
	Unrecorded	10 (2.2)	11 086 (3.9)	0.54	0.28‐1.02	.06	
Age category (years)	<3.0	97 (21.2)	105 557 (37.0)	Base			<.001
	3.0‐<6.0	66 (14.4)	59 813 (21.0)	1.20	0.88‐1.64	.2	
	6.0‐<9.0	65 (14.2)	38 117 (13.4)	1.86	1.36‐2.54	<.001	
	9.0‐<12.0	63 (13.8)	26 999 (9.5)	2.54	1.85‐3.49	<.001	
	12.0‐<15.0	64 (14.0)	22 536 (7.9)	3.09	2.25‐4.24	<.001	
	≥15.0	97 (21.2)	22 239 (7.8)	4.75	3.58‐6.29	<.001	
	Unrecorded	6 (1.3)	9808 (3.4)	0.67	0.29‐1.52	.33	
Adult body weight (kg) (>6 months)	<3.0	16 (3.5)	13 023 (4.6)	Base			.0008
	3.0‐<4.0	59 (12.9)	43 179 (15.2)	1.11	0.64‐1.93	.71	
	4.0‐<5.0	68 (14.9)	53 374 (18.7)	1.04	0.60‐1.79	.9	
	5.0‐<6.0	36 (7.9)	31 580 (11.1)	0.93	0.51‐1.67	.803	
	≥6.0	34 (7.4)	16 660 (5.8)	1.66	0.92‐3.01	.09	
	Unrecorded	245 (53.5)	127 273 (44.6)	1.57	0.94‐2.60	.08	
Sex	Female	240 (52.4)	145 668 (51.1)	Base			.5
	Male	216 (47.2)	136 860 (48.0)	0.96	0.80‐1.15	.7	
	Unrecorded	2 (0.4)	2561 (0.9)	0.47	0.12‐1.91	.3	
Neuter status	Entire	39 (8.5)	40 927 (14.4)	Base			<.001
	Neutered	285 (62.2)	183 783 (64.5)	1.63	1.16‐2.27	.004	
	Unrecorded	134 (29.3)	60 379 (21.2)	2.33	1.63‐3.33	<.001	
Insurance	Noninsured	73 (15.9)	28 652 (10.1)	Base			<.001
	Insured	88 (19.2)	26 636 (9.3)	1.30	0.95‐1.77	.1	
	Unrecorded	297 (64.9)	229 801 (80.6)	0.51	0.39‐0.66	<.001	

*Notes*: Column percentages shown in brackets. N = 285 547.

Abbreviation: CI, confidence interval.

Of the noncase cats with complete data, 29 034/280 966 (10.3%) were purebred, 145 668/282 528 (51.6%) were female, 183 783/224 710 (81.8%) were neutered, 26 636/55 288 (48.2%) were insured and 137 752/274 003 (50.3%) were self‐colored. The most common colors of noncase cats (color information available on 274 003 cats) were black (n = 108 551; 39.6%) and tabby (n = 52 152; 19.0%). The median adult body weight for noncase cats was 4.4 kg (IQR, 3.7‐5.2; range: 1.0‐19.8). The median age for noncase cats on December 31, 2013 was 4.4 years (IQR, 1.7‐9.3; range, 0.0‐28.7; Table 2). Data completeness varied for the variables assessed: sex, 99.1%; breed, 98.6%; age, 96.6%; color, 96.1%; neuter status; 78.8%, body weight; 59.6%, insurance; 19.4%.

### 3.1 Risk factors for RSD in the overall population of cats

Univariable logistic regression modeling identified 7 variables liberally associated with RSD that were further evaluated in multivariable logistic regression modeling: purebred, breed, self‐color, age, adult body weight, neuter status and insurance. The final multivariable model retained 2 risk factors: age, and insurance. No biologically significant interactions were identified in the final model. The final model was not improved by inclusion of the clinic attended as a random effect (rho, 0.008; *P* = .26). The final model showed acceptable model fit (Hosmer‐Lemeshow test statistic, *P* = .14) and discrimination (area under the ROC curve, 0.681). The odds of RSD increased progressively as cats aged. Compared with cats aged <3.0 years, cats aged 9.0 to <12.0 years had 2.23 times the odds (95% CI, 1.62‐3.07; *P* < .001) and cats aged >15.0 years had 4.27 times the odds (95% CI, 3.21‐5.69; *P* < .001). Insured cats had 1.49 (95% CI, 1.09‐2.04; *P* = .01) times the odds of RSD compared with uninsured cats (Table 3).

**TABLE 3:** Final multivariable logistic regression model for risk factors associated with diagnosis of recurrent seizure disorders (RSDs) in cats under primary veterinary care in the UK

Variable	Category	Odds ratio	95% CI	Category *P*‐value	Variable *P*‐value
Age category (years)	<3.0	Base			<.001
	3.0‐<6.0	1.08	0.79‐1.48	.64	
	6.0‐<9.0	1.64	1.20‐2.26	.002	
	9.0‐<12.0	2.23	1.62‐3.07	<.001	
	12.0‐<15.0	2.72	1.98‐3.75	<.001	
	≥15.0	4.27	3.21‐5.69	<.001	
	Unrecorded	0.71	0.31‐1.61	.41	
Insurance	Noninsured	Base			<.001
	Insured	1.49	1.09‐2.04	.012	
	Unrecorded	0.66	0.51‐0.86	.002	

*Notes*: N = 285 547.

Abbreviation: CI, confidence interval.

### 3.2 Risk factors for epilepsy in the overall population of cats

Univariable logistic regression modeling identified 5 variables that were liberally associated with epilepsy and were further evaluated in multivariable logistic regression modeling: breed, age, adult body weight, neuter status and insurance (Table 4). The final multivariable model retained 2 risk factors: age, and insurance. No biologically significant interactions were identified in the final model. The final model was not improved by inclusion of the clinic attended as a random effect (rho, 0.008; *P* < .44). The final model showed acceptable model fit (Hosmer‐Lemeshow test statistic, *P* = .16) and discrimination (area under the ROC curve, 0.700). Compared with cats aged <3.0 years, the odds of epilepsy were higher in all older age groups except for cats aged 12.0 to <15.0 years. Insured cats had 2.38 (95% CI, 1.31‐4.33; *P* = .004) times the odds of epilepsy compared with uninsured cats (Table 5).

**TABLE 4:** Descriptive and univariable logistic regression results for risk factors associated with diagnosis of epilepsy by primary care practitioners in cats under primary veterinary care in the UK

Variable	Category	Case no. (%)	Noncase no. (%)	Odds ratio	95% CI	Category *P*‐value	Variable *P*‐value
Purebred status	Crossbred	103 (90.4)	251 932 (88.4)	Base			.81
	Purebred	11 (9.6)	29 034 (10.2)	0.93	0.50‐1.73	.81	
	Unrecorded	0 (0.0)	4123 (1.5)	…			
Common breeds	Crossbred	103 (90.4)	251 932 (88.4)	Base			.2
	Bengal	1 (0.9)	3340 (1.2)	0.73	0.10‐5.25	.8	
	Birman	1 (0.9)	1145 (0.4)	2.14	0.30‐15.32	.5	
	British Blue	0 (0.0)	1507 (0.5)	…			
	British Short Hair	4 (3.5)	5275 (1.9)	1.85	0.68‐5.04	.23	
	Burmese	2 (1.8)	1496 (0.5)	3.27	0.81‐13.26	.1	
	Burmilla	0 (0.0)	118 (0.0)	…			
	Exotic	2 (1.8)	448 (0.2)	10.92	2.69‐44.38	.001	
	Foreign	0 (0.0)	52 (0.0)	…			
	Maine Coon	0 (0.0)	1967 (0.7)	…			
	Norwegian Forest	0 (0.0)	567 (0.2)	…			
	Other purebreds	0 (0.0)	3664 (1.3)	…			
	Persian	0 (0.0)	3309 (1.2)	…			
	Ragdoll	0 (0.0)	2903 (1.0)	…			
	Russian	0 (0.0)	506 (0.2)	…			
	Siamese	1 (0.9)	2502 (0.9)	0.98	0.14‐7.01	1	
	Siberian	0 (0.0)	235 (0.1)	…			
	Unrecorded	0 (0.0)	4123 (1.5)	…			
Self‐color	Single	55 (48.3)	137 752 (48.3)	Base			.41
	Multiple	57 (50.0)	136 251 (47.8)	1.05	0.72‐1.52	.81	
	Unrecorded	2 (1.7)	11 082 (3.9)	0.45	0.11‐1.85	.27	
Dominant color	Black	53 (46.5)	108 551 (38.1)	Base			.34
	Tabby	19 (16.7)	52 152 (18.3)	0.75	0.44‐1.26	.3	
	Ginger	9 (7.9)	25 902 (9.1)	0.71	0.35‐1.44	.4	
	Tortoiseshell	11 (9.7)	24 508 (8.6)	0.92	0.48‐1.76	.8	
	Gray	2 (1.8)	16 182 (5.7)	0.25	0.06‐1.04	.1	
	White	7 (6.1)	15 357 (5.4)	0.93	0.42‐2.05	.9	
	Blue	3 (2.6)	6219 (2.2)	0.99	0.31‐3.16	1	
	Other color	8 (7.0)	25 132 (8.8)	0.65	0.31‐1.37	.3	
	Unrecorded	2 (1.8)	11 086 (3.9)	0.37	0.09‐1.52	.2	
Age category (years)	<3.0	21 (18.4)	105 557 (37.0)	Base			.001
	3.0‐<6.0	31 (27.2)	59 813 (21.0)	2.61	1.50‐4.53	.001	
	6.0‐<9.0	19 (16.7)	38 117 (13.4)	2.51	1.35‐4.66	.004	
	9.0‐<12.0	17 (14.9)	26 999 (9.5)	3.17	1.67‐6.00	<.001	
	12.0‐<15.0	9 (7.9)	22 536 (7.9)	2.01	0.92‐4.38	.1	
	≥15.0	14 (12.3)	22 239 (7.8)	3.16	1.61‐6.22	.001	
	Unrecorded	3 (2.6)	9808 (3.4)	1.54	0.46‐5.16	.5	
Adult body weight (kg) (>6 months)	<3.0	1 (0.9)	13 023 (4.6)	0.26	0.03‐1.95	.2	.02
	3.0‐<4.0	13 (11.4)	43 179 (15.2)	Base			
	4.0‐<5.0	13 (11.4)	53 374 (18.7)	0.81	0.37‐1.75	.6	
	5.0‐<6.0	15 (13.2)	31 580 (11.1)	1.58	0.75‐3.32	.23	
	≥6.0	9 (7.9)	16 660 (5.8)	1.79	0.77‐4.20	.2	
	Unrecorded	63 (55.3)	127 273 (44.6)	1.64	0.90‐2.99	.103	
Sex	Female	62 (54.4)	145 668 (51.1)	Base			.8
	Male	51 (44.7)	136 860 (48.0)	0.88	0.60‐1.27	.5	
	Unrecorded	1 (0.9)	2561 (0.9)	0.92	0.13‐6.62	.93	
Neuter status	Entire	6 (5.3)	40 927 (14.4)	Base			.001
	Neutered	72 (63.2)	183 783 (64.5)	2.67	1.16‐6.15	.021	
	Unrecorded	36 (31.6)	60 379 (21.2)	4.07	1.71‐9.65	.001	
Insurance	Noninsured	16 (14.0)	28 652 (10.1)	Base			<.001
	Insured	34 (29.8)	26 636 (9.3)	2.29	1.26‐4.14	.006	
	Unrecorded	64 (56.1)	229 801 (80.6)	0.49	0.29‐0.86	.01	

*Notes*: Column percentages shown in brackets. N = 285 203.

Abbreviation: CI, confidence interval.

**TABLE 5:** Final multivariable logistic regression model for risk factors associated with diagnosis of epilepsy by primary‐care practitioners in cats under primary veterinary care in the UK

Variable	Category	Odds ratio	95% CI	Category *P*‐value	Variable *P*‐value
Age category (years)	<3.0	Base			.04
	3.0‐<6.0	2.15	1.22‐3.76	.008	
	6.0‐<9.0	2.01	1.07‐3.76	.03	
	9.0‐<12.0	2.50	1.31‐4.79	.006	
	12.0‐<15.0	1.61	0.73‐3.55	.234	
	≥15.0	2.69	1.36‐5.34	.005	
	Unrecorded	1.74	0.52‐5.85	.4	
Insurance	Noninsured	Base			<.001
	Insured	2.38	1.31‐4.33	.004	
	Unrecorded	0.59	0.34‐1.04	.07	

*Notes*: N = 285 203.

Abbreviation: CI, confidence interval.

### 3.3 Risk factors for epilepsy among cats with RSD

Multivariable logistic regression modeling identified age as the only factor associated with classification of epilepsy (114 epilepsy cases) among the subset of 458 cats with RSD. Cats aged 3.0 to <6.0 years had 3.32 (95% CI, 1.66‐6.67; *P* = .001) times the odds of RSD compared with cats aged <3.0 years. Insurance was retained as a confounder in the final model (Table 6).

**TABLE 6:** Final multivariable logistic regression model for risk factors associated with classification as epilepsy by primary‐care practitioners among cats diagnosed with recurrent seizure disorders (RSDs) under primary veterinary care in the UK

Variable	Category	Odds ratio	95% CI	Category *P*‐value	Variable *P*‐value
Age category (years)	<3.0	Base			.005
	3.0‐<6.0	3.32	1.66‐6.67	.001	
	6.0‐<9.0	1.53	0.74‐3.18	.2	
	9.0‐<12.0	1.38	0.65‐2.91	.4	
	12.0‐<15.0	0.61	0.26‐1.45	.3	
	≥15.0	0.61	0.28‐1.45	.3	
	Unrecorded	4.72	0.87‐25.41	.1	
Insurance	Noninsured	Base			<.001
	Insured	1.88	0.91‐3.90	.1	
	Unrecorded	0.77	0.40‐1.47	.42	

*Notes*: N = 458.

Abbreviation: CI, confidence interval.

## 4 DISCUSSION

Ours is the first study to explore RSD and epilepsy in cats by analyzing data from a multicenter primary care research database in the UK. Our study of 285 547 cats attending UK primary care practices in 2013 reports a 1‐year period prevalence of 0.16% for RSD and 0.04% for epilepsy as classified by primary care veterinary practitioners. These results confirm that although RSD are less frequently diagnosed in cats than in dogs,^7^, ^8^, ^37^ RSD are a relatively common clinical presentation for cats evaluated by veterinary practitioners, which is consistent with previous reports.^38^, ^39^ Numerically, despite the relatively low prevalence of these disorders, an estimated 10.9 million cats are owned in the UK with 24% of UK adults owning a cat,^40^ which equates to a substantial number of potentially affected cats in the UK. Our findings are substantially lower than the 2.1% and 3.5% prevalences of seizures previously reported in referral feline populations.^3^, ^4^ This difference likely reflects inherent differences in caseloads between primary care and referral practices,^6^ and mirrors a discrepancy described in Germany where the primary care period prevalence of dogs presenting with seizures was 0.43% compared to 1.78% in a referral population.^41^ Considering the differences between cats and dogs, although the prevalence for RSD in cats is considerably lower than the prevalence in dogs with at least 1 seizure reported previously,^8^ the inclusion criteria for RSD in our study required cats to have had at least 2 seizure events separated by at least 24 hours, an epilepsy diagnosis, or treatment using ASD. Given that toxicity and metabolic disturbances (reactive seizures) are responsible for many single seizure events,^3^, ^38^, ^39^ this situation may account for a proportion of the differences reported between the species. Additionally, recognition of seizures in cats often is complicated by the heterogenous semiology of the condition in this species,^4^, ^13^, ^42^, ^43^ such that the true proportion of cats with RSD may be higher than that reported by owners. Furthermore, compared to dogs, many UK cats spend a considerable proportion of their time outdoors, where owners are less likely to witness seizure events.^39^

Despite substantial progress in recent decades on the classification of neurological diseases in companion animals^5^ along with efforts to formally define internationally‐accepted diagnostic lexicons and definitions,^19^ reluctance remains for primary care practitioners to evaluate and identify their neurological clinical caseloads.^44^ This reluctance for standardized diagnosis recording means that many primary care cases are recorded using phenotypic signature (ie, seizure disorder) rather than a formal diagnosis term (eg, epilepsy). This phenomenon was highlighted in a recent study of seizure disorders in dogs presenting to primary care clinics where only 10.7% of idiopathic epilepsy cases that met IVETF criteria were recorded as epilepsy or idiopathic epilepsy in the clinical records.^45^ One impact for scientific research arising from this tendency toward informal diagnosis recording is that studies that rely on formally‐recorded diagnosis terms are likely to substantially underreport the true frequency of these disorders. Consequently, and in an effort to include all true cases in our study, we chose not to rely on formal diagnosis terms such as epilepsy or idiopathic epilepsy as recorded in the clinical notes but instead to focus more on a phenotypic signature that would identify cats with RSD.

A formal diagnosis of epilepsy was recorded by the first opinion veterinary practitioner for 114 (24.89%) of the 458 RSD cases, although it is likely that many more of these cases met the internationally‐agreed definition for epilepsy despite not being recorded as such by the attending veterinarians.^19^ The precise clinical criteria applied for the epilepsy diagnoses recorded in the clinical records was not explored in our study, but these could be the topic of future research exploring how closely primary care clinicians apply IVETF diagnosis guidelines.^44^ It is likely that a final pathophysiological rationale for the disorder was not reached in many of the cases that were not recorded as epilepsy, and therefore the attending veterinarians recorded the condition based on its phenotypic signature rather than spuriously recording a biomedical diagnosis term that they could not be confident was correct. Our study applied the diagnosis term “epilepsy” to include subclassifications including idiopathic and structural epilepsy as well as epilepsy of unknown cause.^19^

Diagnosis of “epilepsy” in veterinary medicine classically requires ≥2 seizures that are 24 hours apart and cases without a known underlying cause generally are recorded as “idiopathic epilepsy.”^46^ As with all idiopathic conditions, idiopathic epilepsy is a diagnosis of exclusion, and therefore relies on the clinical acumen and resources available to the relevant veterinary teams.^47^ In dogs aged 6 months to 6 years with normal interictal neurological examination, lack of clinically relevant abnormalities on routine blood serum biochemical and urinalysis tests with typical seizure presentation is consistent with Tier I level of diagnostic confidence.^44^ A similar consensus view on diagnostic confidence for cats currently is not available. In cats, idiopathic epilepsy historically has been considered relatively rare, but in recent years more investigators have used the term routinely for cats and reported that up to 57% of cats with epilepsy might be classified as having idiopathic epilepsy.^13^ It is conceivable that general veterinary practitioners may feel reluctant to formally diagnose epilepsy or idiopathic epilepsy in cats because of a combination of factors, including their limited confidence in performing a complete neurological examination in cats, the longstanding traditional belief that cats do not commonly have idiopathic epilepsy, and a belief that access to advanced imaging is essential to exclude other causes. Epilepsy was discussed as a possible diagnosis in a further 69 cats in our study, which could increase the proportion of RSD cases with a diagnosis of epilepsy to 183/458 cats (39.95%), although this figure could be substantially lower because cats >7 years of age are more likely to have structural epilepsy.^43^ Cats with RSD cats that have a normal interictal neurological examination not infrequently are identified as having structural epilepsy based on advanced imaging, with 12.2% having clinically relevant magnetic resonance imaging (MRI) abnormalities reported in 1 study.^48^ This proportion is similar to the 11.8% of clinically relevant MRI findings reported in dogs with normal interictal neurological examinations.^49^ Thus, it is likely that some cats diagnosed with epilepsy in our study may have had an unrecognized structural cause that advanced imaging would have elucidated.

The median age at first recorded seizure event for RSD overall in our study was 8.9 years, which was comparatively higher than the median age of 6.4 years at first recorded seizure event for the subset of cats with RSD that we recorded as having epilepsy. Results of the risk factor modeling highlight the age group that is most likely to be diagnosed with epilepsy from among the RSD caseloads: cats aged 3 to 6 years had 3.32 times the odds of diagnosis compared with cats aged <3 years old. This tendency to diagnose epilepsy in younger cats among RSD caseloads is consistent with most previous studies, which reported cats diagnosed with epilepsy as younger than cats with structural causes. The reported ages of 3.4 to 4.6 years for epilepsy are lower than the 8.1 to 9.2 years reported for cats with structural causes.^3^, ^4^, ^20^, ^43^, ^48^, ^50^ Compared with cats <3 years of age, the odds of a diagnosis of epilepsy was higher in all older age groups (except for cats aged 12‐15 years). Therefore, although 3 to 6 years may be the peak time at which most cases of idiopathic epilepsy in cats are diagnosed, this condition should not be excluded for diagnosis in older animals simply because of their age. However, the odds of diagnosis of epilepsy in both the overall population and among the RSD caseloads suggest that cats <3 years of age have lower odds of diagnosis with epilepsy. These findings for the age at which epilepsy is most likely to affect cats can serve as a benchmark for practitioners when prioritizing differential causes of RSD in cats. Furthermore, this information may assist in the future development of guidelines in cats for the classification and diagnosis of idiopathic epilepsy.

Although the odds of diagnosis specifically with epilepsy were highest in cats aged 3 to 6 years, the odds of diagnosis with RSD in general increased progressively with age. Structural causes of RSD are reported in 40% to 70% of affected cats.^13^ The RSD caseload in our study likely includes several underlying conditions, including neoplastic and vascular disorders that predominantly affect older animals.^9^, ^43^ Additionally, although recognized cases of reactive seizures were excluded from our study, some may have remained given that not all cases were comprehensively evaluated by complete serum biochemistry, urinalysis and blood pressure measurement. Because reactive seizures are also predominantly caused by conditions that preferentially affect older animals,^39^ potential retention of some misclassified reactive seizure cases in our study may have increased the apparent probability of RSD in older cats. Seizures therefore should be considered as a common cause of paroxysmal events in cats, particularly with increasing age. This information may offer primary practitioners evidence with which to advise medical and possibly advanced imaging studies in cases in which seizures are suspected.

Insured cats had 1.49 times the odds of diagnosis with RSD compared with uninsured cats in our study. Animals that are insured are more likely to be presented to veterinary practices for investigation of medical problems because financial barriers to presentation are decreased.^51^ Although it is also possible that a small proportion of cats may have become insured because their owners suspected a health problem, it is more likely that the higher diagnosis rates in insured cats reflect greater awareness of disease in insured cats rather than truly higher rates of inherent disease. The association between insurance and diagnosis was even higher for epilepsy, with insured cats showing 2.38 times the odds of a diagnosis of epilepsy compared with uninsured cats. Similar associations with increased diagnosis in insured animals have been reported previously for several disorders including diabetes mellitus in cats (×2.0),^52^ lipoma in dogs (×1.78),^53^ hyperadrenocorticism in dogs (×4.0),^54^ and corneal ulceration in dogs (×1.6).^55^ This insurance bias must be considered carefully when generalizing the results of studies that are based entirely on data from insured animals.^37^ We did not report the proportion of animals referred for specialist management or that underwent advanced imaging, but it would have been useful to know these because a normal brain MRI and cerebrospinal fluid (CSF) analysis would increase confidence in a diagnosis of idiopathic epilepsy and insured animals are more likely to undergo this level of diagnostic evaluation.

Our findings do not support an association between breed and RSD in cats. The prevalence of RSD was not statistically different between crossbred cats (0.17%) and purebred cats (0.13%, *P* = .16). Breeds in our study with the highest RSD prevalence were Foreign (breed prevalence, 1.89%) and Burmilla (0.84%), but these results should be interpreted with caution because only 1 affected individual was represented in each of these breeds. The relatively low proportion of individual purebred cat breeds in the UK means that our study was grossly underpowered to evaluate breed as a risk factor. Given that the demographic data reported in our study show that crossbred cats comprise the majority of the UK pet cat population, this factor will always make it challenging to achieve good statistical power for breed comparisons in cats, even with access to increasingly large research datasets over time.^25^, ^26^, ^27^ However, ownership of purebred cats currently is increasing, which may facilitate breed‐based research in cats in the future.^56^

Specifically in relation to epilepsy, the results of univariable analysis in our study indicated that Burmese, Birman, and British Short Hair cats to have 3.27, 2.14, and 1.85 times the odds, respectively, of epilepsy compared to crossbreed cats, but the breed variable was not retained on multivariable analysis. The low counts of affected animals of these breeds in our study precludes firm conclusions from being drawn about breed risk, but these breeds perhaps should be considered specifically in future explorations of genetic factors for epilepsy of cats. A previous referral study in the UK reported that pedigreed cats had 5.68 times the odds of epilepsy compared with nonpedigreed cats.^43^ However, unlike the situation in dogs, specific breeds have not yet been linked to epilepsy in cats, and genetic epilepsy only has been reported in study colonies of cats.^11^, ^17^, ^18^ A study of audiogenic reflex seizures in cats reported that a large number of affected cats were Birmans.^57^ Audiogenic reflex seizures are proposed to have a genetic basis despite their geriatric onset and possibly represent a genetic type of epilepsy in cats.^57^ Further investigation of genetic causes of epilepsy in cats would benefit from similar development of rigorous diagnostic criteria of epilepsy of unknown cause in cats, as has been achieved in dogs.^5^, ^19^

No evidence was found of an association between sex and either RSD or epilepsy in cats in our study. This finding is consistent with previous studies in cats.^3^, ^4^ In dogs, males may have increased odds of developing seizure disorders,^7^, ^8^, ^58^ but this observation is complicated by effects of neutering as well as different sex predisposition to seizures among specific dog breeds.^58^, ^59^

Despite the large numbers of cats affected by RSD in our study, no antiseizure medications currently are licensed for use in cats in the UK. Several studies have shown that cats can respond well to medical treatment and furthermore that stopping treatment leads to recurrence of seizure activity in up to 75% of affected cats.^13^, ^60^ Our findings should prompt manufacturers to consider steps to undertake the licensing of antiseizure medications in cats and encourage practitioners to consider medical treatment of affected animals.

Our study had some limitations. Despite there being an estimated 1 million more owned cats than dogs in the UK,^40^ far fewer cats are registered at veterinary practices than dogs.^26^, ^61^ Although our study aimed to provide reliable estimates for the prevalence and demographics of RSD‐affected cats, many owned cats are not under routine veterinary care and thus our results should be interpreted with caution and not generalized. Barriers to regular veterinary presentation of cats may include lower numbers of insured cats than dogs (ie, 7% vs 19% of animals attending VetCompass participating practices) and perceived difficulties in medicating cats. Diagnosis of epilepsy in our study relied on the opinion of the primary care practitioner rather than the formal classification guidelines as laid out by IVETF, which were published after data collection for our study.^5^, ^19^

Only fair to moderate interobserver agreement has been found between neurologists and nonspecialists when diagnosing seizure activity in dogs and cats^15^ and, as discussed above, poor concordance has been shown between primary care and retrospective classification of epilepsy cases in dogs.^45^ Improvement in future studies using similar datasets would include extraction of information pertaining to ancillary testing so as to categorize animals into an IVEFT Tier 1 level of confidence of diagnosis of epilepsy. In addition to the clinical acumen of the individual veterinary practitioner and the available diagnostic tests, an accurate description of events by owners is also essential to generate the initial index of suspicion of seizure activity,^62^ but such ability is likely to vary substantially among owners.

## 5 CONCLUSION

Our large retrospective study using a multicenter primary practice dataset found a 0.16% prevalence of RSD in cats, highlighting that these disorders are not uncommon in this species. Recurrent seizure disorders increase in prevalence with increasing age. No evidence was found for sex and breed associations with RSD in cats, which may reflect multiple etiologies in this species. Epilepsy was recorded in 0.04% of cats by primary care practitioners, with cats aged 3 to 6 years being the most likely age group of cats with RSD to be diagnosed with epilepsy. Future studies using expanded datasets are needed to fully explore questions related to semiology and clinical outcomes. The veterinary neurology community should take efforts to better define the term “epilepsy” in cats and develop diagnostic criteria.

## CONFLICT OF INTEREST DECLARATION

Holger Volk in last 5 years served as contract researcher for: Nestle 2012‐2014 and 2017‐2020—dietary modification of epilepsy in dogs; Boehringer Ingelheim 2014‐2015—investigating the effects of imepitoin behavioral, physiologic and owner reported indicators of anxiety in dogs treated for idiopathic epilepsy; CASE BBSRC PhD studentship 2012‐2016—metabolic profiling of epilepsy in dogs; American Kennel Club American Health Foundation, 2016‐2020—Investigating the Effect of a Ketogenic Medium Chain Triglycerides Supplement on the treatment of Canine Idiopathic Epilepsy and its behavioral comorbidities; BBSRC 2017‐2020—Investigating the relationship between epilepsy, drug‐resistance and affective disorders in the domestic dog BB/P001874/1; provided continuous education for veterinary surgeons sponsored by Nestle, Boehringer Ingelheim and Virbac in the last 5 years.

Dan O'Neill was the supervisor on a study of seizures in dogs in 2018 supported by Bayer.

None of these COI relate to feline seizure disorders or epilepsy. The authors did not allow any bias from any of these COI to affect the design, analysis or writing of the current paper.

## OFF‐LABEL ANTIMICROBIAL DECLARATION

Authors declare no off‐label use of antimicrobials.

## INSTITUTIONAL ANIMAL CARE AND USE COMMITTEE (IACUC) OR OTHER APPROVAL DECLARATION

Authors declare no IACUC or other approval was needed.

## HUMAN ETHICS APPROVAL DECLARATION

Authors declare human ethics approval was not needed for this study.
